# Enhancing senior high school student engagement and academic performance using an inclusive and scalable inquiry-based program

**DOI:** 10.1038/s41539-020-00076-2

**Published:** 2020-12-02

**Authors:** Locke Davenport Huyer, Neal I. Callaghan, Sara Dicks, Edward Scherer, Andrey I. Shukalyuk, Margaret Jou, Dawn M. Kilkenny

**Affiliations:** 1 Institute of Biomedical Engineering, University of Toronto, Toronto, ON Canada; 2 Chemical Engineering and Applied Chemistry, University of Toronto, Toronto, ON Canada; 3 Translational Biology and Engineering Program, Ted Rogers Centre for Heart Research, University of Toronto, Toronto, ON Canada; 4 George Harvey Collegiate Institute, Toronto District School Board, Toronto, ON Canada; 5 Institute for Studies in Transdisciplinary Engineering Education & Practice, University of Toronto, Toronto, ON Canada

**Keywords:** Education, Education

## Abstract

The multi-disciplinary nature of science, technology, engineering, and math (STEM) careers often renders difficulty for high school students navigating from classroom knowledge to post-secondary pursuits. Discrepancies between the knowledge-based high school learning approach and the experiential approach of future studies leaves some students disillusioned by STEM. We present *Discovery*, a term-long inquiry-focused learning model delivered by STEM graduate students in collaboration with high school teachers, in the context of biomedical engineering. Entire classes of high school STEM students representing diverse cultural and socioeconomic backgrounds engaged in iterative, problem-based learning designed to emphasize critical thinking concomitantly within the secondary school and university environments. Assessment of grades and survey data suggested positive impact of this learning model on students’ STEM interests and engagement, notably in under-performing cohorts, as well as repeating cohorts that engage in the program on more than one occasion. *Discovery* presents a scalable platform that stimulates persistence in STEM learning, providing valuable learning opportunities and capturing cohorts of students that might otherwise be under-engaged in STEM.

## Introduction

High school students with diverse STEM interests often struggle to understand the STEM experience outside the classroom^1^. The multi-disciplinary nature of many career fields can foster a challenge for students in their decision to enroll in appropriate high school courses while maintaining persistence in study, particularly when these courses are not mandatory^2^. Furthermore, this challenge is amplified by the known discrepancy between the knowledge-based learning approach common in high schools and the experiential, mastery-based approaches afforded by the subsequent undergraduate model^3^. In the latter, focused classes, interdisciplinary concepts, and laboratory experiences allow for the application of accumulated knowledge, practice in problem solving, and development of both general and technical skills^4^. Such immersive cooperative learning environments are difficult to establish in the secondary school setting and high school teachers often struggle to implement within their classroom^5^. As such, high school students may become disillusioned before graduation and never experience an enriched learning environment, despite their inherent interests in STEM^6^.

It cannot be argued that early introduction to varied math and science disciplines throughout high school is vital if students are to pursue STEM fields, especially within engineering^7^. However, the majority of literature focused on student interest and retention in STEM highlights outcomes in US high school learning environments, where the sciences are often subject-specific from the onset of enrollment^8^. In contrast, students in the Ontario (Canada) high school system are required to complete Level 1 and 2 core courses in science and math during Grades 9 and 10; these courses are offered as ‘applied’ or ‘academic’ versions and present broad topics of content^9^. It is not until Levels 3 and 4 (generally Grades 11 and 12, respectively) that STEM classes become subject-specific (i.e., Biology, Chemistry, and/or Physics) and are offered as “university”, “college”, or “mixed” versions, designed to best prepare students for their desired post-secondary pursuits^9^. Given that Levels 3 and 4 science courses are not mandatory for graduation, enrollment identifies an innate student interest in continued learning. Furthermore, engagement in these post-secondary preparatory courses is also dependent upon achieving successful grades in preceding courses, but as curriculum becomes more subject-specific, students often yield lower degrees of success in achieving course credit^2^. Therefore, it is imperative that learning supports are best focused on ensuring that those students with an innate interest are able to achieve success in learning.

When given opportunity and focused support, high school students are capable of successfully completing rigorous programs at STEM-focused schools^10^. Specialized STEM schools have existed in the US for over 100 years; generally, students are admitted after their sophomore year of high school experience (equivalent to Grade 10) based on standardized test scores, essays, portfolios, references, and/or interviews^11^. Common elements to this learning framework include a diverse array of advanced STEM courses, paired with opportunities to engage in and disseminate cutting-edge research^12^. Therein, said research experience is inherently based in the processes of critical thinking, problem solving, and collaboration. This learning framework supports translation of core curricular concepts to practice and is fundamental in allowing students to develop better understanding and appreciation of STEM career fields.

Despite the described positive attributes, many students do not have the ability or resources to engage within STEM-focused schools, particularly given that they are not prevalent across Canada, and other countries across the world. Consequently, many public institutions support the idea that post-secondary led engineering education programs are effective ways to expose high school students to engineering education and relevant career options, and also increase engineering awareness^13^. Although singular class field trips are used extensively to accomplish such programs, these may not allow immersive experiences for application of knowledge and practice of skills that are proven to impact long-term learning and influence career choices^14,15^. Longer-term immersive research experiences, such as after-school programs or summer camps, have shown successful at recruiting students into STEM degree programs and careers, where longevity of experience helps foster self-determination and interest-led, inquiry-based projects^4,16–19^.

Such activities convey the elements that are suggested to make a post-secondary led high school education programs successful: hands-on experience, self-motivated learning, real-life application, immediate feedback, and problem-based projects^20,21^. In combination with immersion in university teaching facilities, learning is authentic and relevant, similar to the STEM school-focused framework, and consequently representative of an experience found in actual STEM practice^22^. These outcomes may further be a consequence of student engagement and attitude: Brown et al. studied the relationships between STEM curriculum and student attitudes, and found the latter played a more important role in intention to persist in STEM when compared to self-efficacy^23^. This is interesting given that student self-efficacy has been identified to influence ‘motivation, persistence, and determination’ in overcoming challenges in a career pathway^24^. Taken together, this suggests that creation and delivery of modern, exciting curriculum that supports positive student attitudes is fundamental to engage and retain students in STEM programs.

Supported by the outcomes of identified effective learning strategies, University of Toronto (U of T) graduate trainees created a novel high school education program *Discovery*, to develop a comfortable yet stimulating environment of inquiry-focused iterative learning for senior high school students (Grades 11 & 12; Levels 3 & 4) at non-specialized schools. Built in strong collaboration with science teachers from George Harvey Collegiate Institute (Toronto District School Board), *Discovery* stimulates application of STEM concepts within a unique term-long applied curriculum delivered iteratively within both U of T undergraduate teaching facilities and collaborating high school classrooms^25^. Based on the volume of medically-themed news and entertainment that is communicated to the population at large, the rapidly-growing and diverse field of biomedical engineering (BME) were considered an ideal program context^26^. In its definition, BME necessitates cross-disciplinary STEM knowledge focused on the betterment of human health, wherein *Discovery* facilitates broadening student perspective through engaging inquiry-based projects. Importantly, *Discovery* allows all students within a class cohort to work together with their classroom teacher, stimulating continued development of a relevant learning community that is deemed essential for meaningful context and important for transforming student perspectives and understandings^27,28^. Multiple studies support the concept that relevant learning communities improve student attitudes towards learning, significantly increasing student motivation in STEM courses, and consequently improving the overall learning experience^29^. Learning communities, such as that provided by *Discovery*, also promote the formation of self-supporting groups, greater active involvement in class, and higher persistence rates for participating students^30^.

The objective of *Discovery*, through structure and dissemination, is to engage senior high school science students in challenging, inquiry-based practical BME activities as a mechanism to stimulate comprehension of STEM curriculum application to real-world concepts. Consequent focus is placed on critical thinking skill development through an atmosphere of perseverance in ambiguity, something not common in a secondary school knowledge-focused delivery but highly relevant in post-secondary STEM education strategies. Herein, we describe the observed impact of the differential project-based learning environment of *Discovery* on student performance and engagement. We identify the value of an inquiry-focused learning model that is tangible for students who struggle in a knowledge-focused delivery structure, where engagement in conceptual critical thinking in the relevant subject area stimulates student interest, attitudes, and resulting academic performance. Assessment of study outcomes suggests that when provided with a differential learning opportunity, student performance and interest in STEM increased. Consequently, *Discovery* provides an effective teaching and learning framework within a non-specialized school that motivates students, provides opportunity for critical thinking and problem-solving practice, and better prepares them for persistence in future STEM programs.

## Results

### Program delivery

**Fig. 1:**
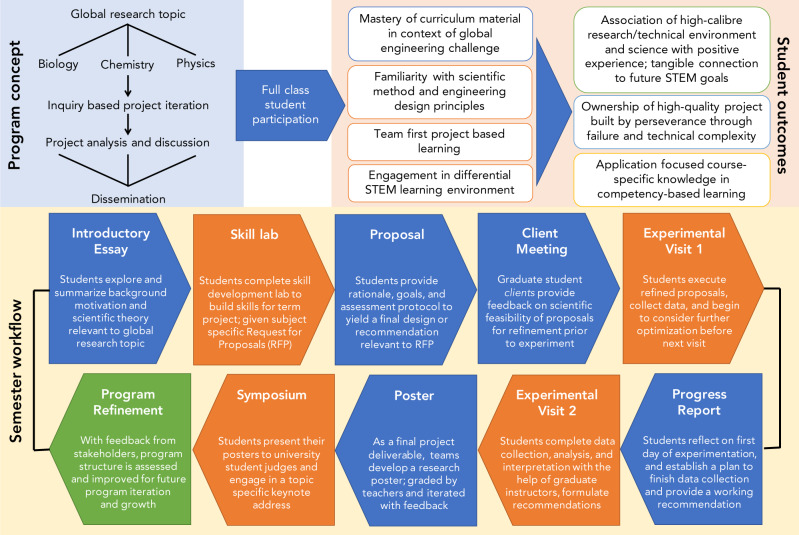
Structure and rationale underlying the *Discovery* framework. The general program concept (blue background; *top left*) highlights a global research topic examined through student dissemination of subject-specific research questions, yielding multifaceted student outcomes (orange background; *top right*). Each program term (term workflow, yellow background; *bottom panel*), students work on program deliverables in class (blue), iterate experimental outcomes within university facilities (orange), and are assessed accordingly at numerous deliverables in an inquiry-focused learning model.

The outcomes of the current study result from execution of *Discovery* over five independent academic terms as a collaboration between Institute of Biomedical Engineering (graduate students, faculty, and support staff) and George Harvey Collegiate Institute (science teachers and administration) stakeholders. Each term, the program allowed senior secondary STEM students (Grades 11 and 12) opportunity to engage in a novel project-based learning environment. The program structure uses the problem-based engineering capstone framework as a tool of inquiry-focused learning objectives, motivated by a central BME global research topic, with research questions that are inter-related but specific to the curriculum of each STEM course subject (Fig. 1). Over each 12-week term, students worked in teams (3–4 students) within their class cohorts to execute projects with the guidance of U of T trainees (*Discovery* instructors) and their own high school teacher(s). Student experimental work was conducted in U of T teaching facilities relevant to the research study of interest (i.e., Biology and Chemistry-based projects executed within Undergraduate Teaching Laboratories; Physics projects executed within Undergraduate Design Studios). Students were introduced to relevant techniques and safety procedures in advance of iterative experimentation. Importantly, this experience served as a course term project for students, who were assessed at several points throughout the program for performance in an inquiry-focused environment as well as within the regular classroom (Fig. 1). To instill the atmosphere of STEM, student teams delivered their outcomes in research poster format at a final symposium, sharing their results and recommendations with other post-secondary students, faculty, and community in an open environment.

Over the course of five terms there were 268 instances of tracked student participation, representing 170 individual students. Specifically, 94 students participated during only one term of programming, 57 students participated in two terms, 16 students participated in three terms, and 3 students participated in four terms. Multiple instances of participation represent students that enrol in more than one STEM class during their senior years of high school, or who participated in Grade 11 and subsequently Grade 12. Students were surveyed before and after each term to assess program effects on STEM interest and engagement. All grade-based assessments were performed by high school teachers for their respective STEM class cohorts using consistent grading rubrics and assignment structure. Here, we discuss the outcomes of student involvement in this experiential curriculum model.

### Student performance and engagement

**Fig. 2:**
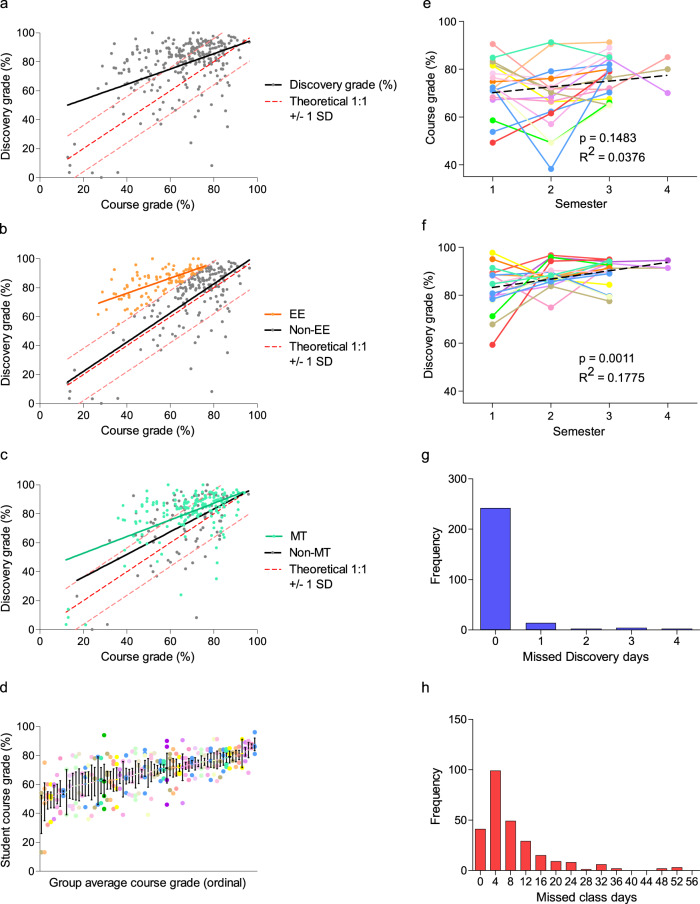
Student aggregate performance in *Discovery* and identification of student subsets. **a** Linear regression of student grades reveals a significant correlation (*p* = 0.0009) between *Discovery* performance and final course grade less the *Discovery* contribution to grade, as assessed by teachers. The dashed red line and intervals represent the theoretical 1:1 correlation between *Discovery* and course grades and standard deviation of the *Discovery*-course grade differential, respectively. **b**, **c** Identification of subgroups of interest, Exceeds Expectations (EE; *N* = 99, *orange*) who were ≥+1 SD in *Discovery*-course grade differential and Multi-Term (MT; *N* = 174, *teal*), of which *N* = 65 students were present in both subgroups. **d** Students tended to self-assemble in working groups according to their final course performance; data presented as mean ± SEM. **e** For MT students participating at least 3 terms in *Discovery*, there was no significant correlation between course grade and time, while (**f**) there was a significant correlation between *Discovery* grade and cumulative terms in the program. Histograms of total absences per student in (**g**) *Discovery* and (**h**) class (binned by 4 days to be equivalent in time to a single *Discovery* absence).

Student grades were assigned, collected, and anonymized by teachers for each *Discovery* deliverable (background essay, client meeting, proposal, progress report, poster, and final presentation). Teachers anonymized collective *Discovery* grades, the component deliverable grades thereof, final course grades, attendance in class and during programming, as well as incomplete classroom assignments, for comparative study purposes. Students performed significantly higher in their cumulative *Discovery* grade than in their cumulative classroom grade (final course grade less the *Discovery* contribution; *p* < 0.0001). Nevertheless, there was a highly significant correlation (*p* < 0.0001) observed between the grade representing combined *Discovery* deliverables and the final course grade (Fig. 2a). Further examination of the full dataset revealed two student cohorts of interest: the “Exceeds Expectations” (EE) subset (defined as those students who achieved ≥1 SD [18.0%] grade differential in *Discovery* over their final course grade; *N* = 99 instances), and the “Multiple Term” (MT) subset (defined as those students who participated in *Discovery* more than once; 76 individual students that collectively accounted for 174 single terms of assessment out of the 268 total student-terms delivered) (Fig. 2b, c). These subsets were not unrelated; 46 individual students who had multiple experiences (60.5% of total MTs) exhibited at least one occasion in achieving a ≥18.0% grade differential. As students participated in group work, there was concern that lower-performing students might negatively influence the *Discovery* grade of higher-performing students (or vice versa). However, students were observed to self-organize into groups where all individuals received similar final overall course grades (Fig. 2d), thereby alleviating these concerns.

The benefits experienced by MT students seemed progressive; MT students that participated in 3 or 4 terms (*N* = 16 and 3, *respectively*) showed no significant increase by linear regression in their course grade over time (*p* = 0.15, Fig. 2e), but did show a significant increase in their *Discovery* grades (*p* = 0.0011, Fig. 2f). Finally, students demonstrated excellent *Discovery* attendance; at least 91% of participants attended all *Discovery* sessions in a given term (Fig. 2g). In contrast, class attendance rates reveal a much wider distribution where 60.8% (163 out of 268 students) missed more than 4 classes (equivalent in learning time to one *Discovery* session) and 14.6% (39 out of 268 students) missed 16 or more classes (equivalent in learning time to an entire program of *Discovery*) in a term (Fig. 2h).

**Fig. 3:**
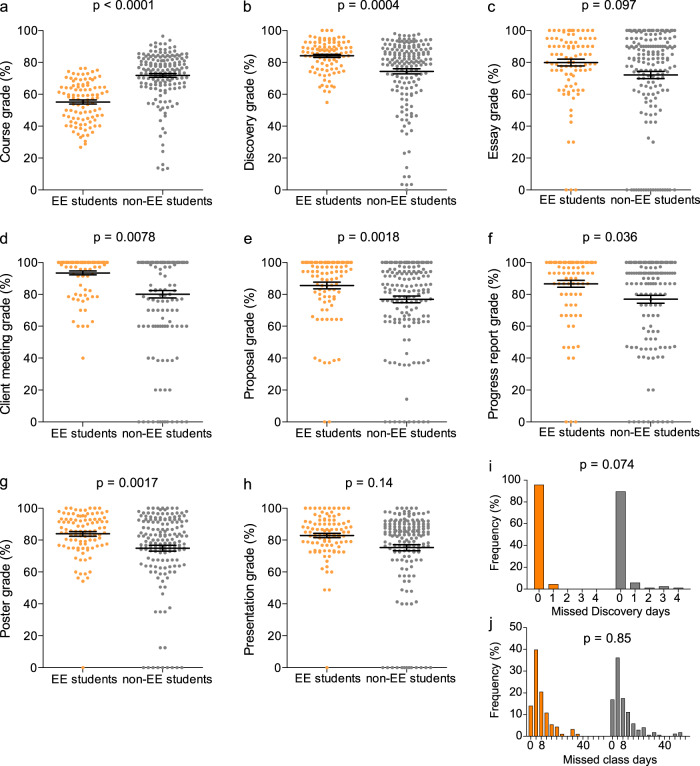
Performance of “Exceeds Expectations” student subset. The “Exceeds Expectations” (EE) subset of students (defined as those who received a combined *Discovery* grade ≥1 SD (18.0%) higher than their final course grade) performed (**a**) lower on their final course grade and (**b**) higher in the *Discovery* program as a whole when compared to their classmates. **d**–**h** EE students received significantly higher grades on each *Discovery* deliverable than their classmates, except for their (**c**) introductory essays and (**h**) final presentations. The EE subset also tended (**i**) to have a higher relative rate of attendance during *Discovery* sessions but no difference in (**j**) classroom attendance. *N* = 99 EE students and 169 non-EE students (268 total). Grade data expressed as mean ± SEM.

*Discovery* EE students (Fig. 3), roughly by definition, obtained lower course grades (*p* < 0.0001, Fig. 3a) and higher final *Discovery* grades (*p* = 0.0004, Fig. 3b) than non-EE students. This cohort of students exhibited program grades higher than classmates (Fig. 3c–h); these differences were significant in every category with the exception of essays, where they outperformed to a significantly lesser degree (*p* = 0.097; Fig. 3c). There was no statistically significant difference in EE vs. non-EE student classroom attendance (*p* = 0.85; Fig. 3i, j). There were only four single day absences in *Discovery* within the EE subset; however, this difference was not statistically significant (*p* = 0.074).

**Fig. 4:**
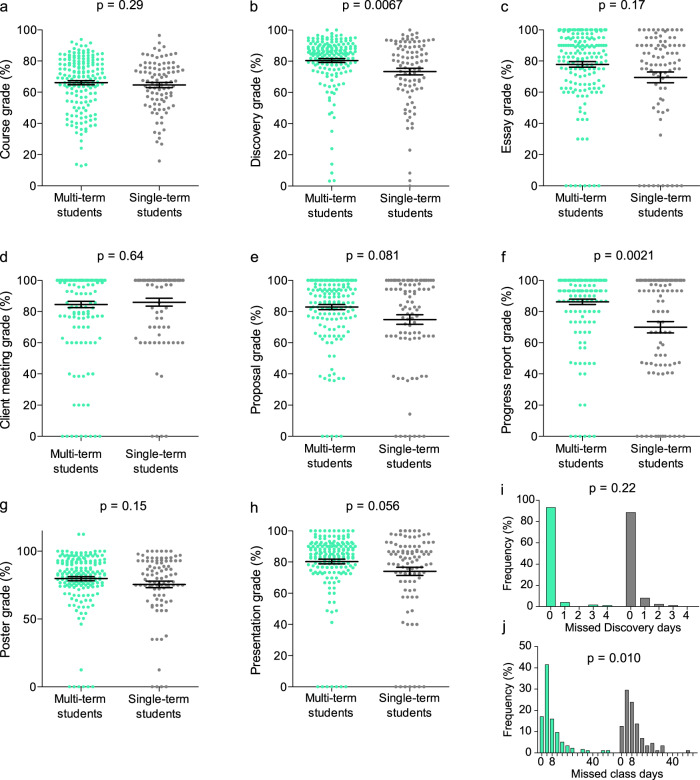
Performance of “multi-term” student subset. The “multi-term” (MT) subset of students (defined as having attended more than one term of *Discovery*) demonstrated favorable performance in *Discovery*, (**a**) showing no difference in course grade compared to single-term students, but (**b** outperforming them in final *Discovery* grade. Independent of the number of times participating in *Discovery*, MT students did not score significantly differently on their (**c**) essay, (**d**) client meeting, or (**g**) poster. They tended to outperform their single-term classmates on the (**e**) proposal and (**h**) final presentation and scored significantly higher on their (**f**) progress report. MT students showed no statistical difference in (**i**) *Discovery* attendance but did show (**j**) higher rates of classroom attendance than single-term students. *N* = 174 MT instances of student participation (76 individual students) and 94 single-term students. Grade data expressed as mean ± SEM.

**Fig. 5:**
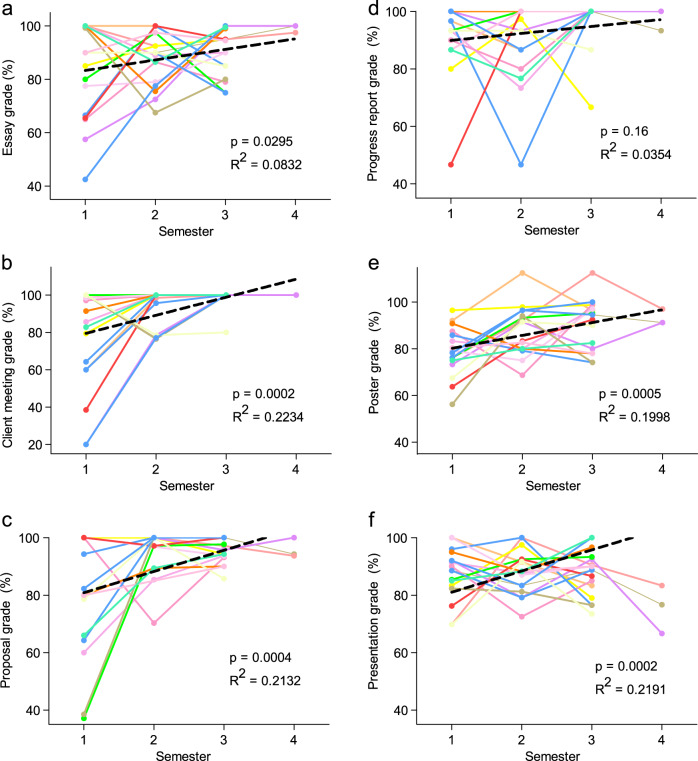
Academic performance of MT student participants improves in *Discovery* deliverables over multiple terms of participation. Longitudinal assessment of a subset of MT student participants that participated in three (*N* = 16) or four (*N* = 3) terms presents a significant trend of improvement in their (**a**) essay, (**b**) client meeting, (**c**) proposal, (**e**) poster, and (**f**) presentation grade. **d** Progress report grades present a trend in improvement but demonstrate strong performance in all terms, limiting potential for student improvement. Grade data are presented as individual student performance; each student is represented by one color; data is fitted with a linear trendline (black).

*Discovery* MT students (Fig. 4), although not receiving significantly higher grades in class than students participating in the program only one time (*p* = 0.29, Fig. 4a), were observed to obtain higher final *Discovery* grades than single-term students (*p* = 0.0067, Fig. 4b). Although trends were less pronounced for individual MT student deliverables (Fig. 4c–h), this student group performed significantly better on the progress report (*p* = 0.0021; Fig. 4f). Trends of higher performance were observed for initial proposals and final presentations (*p* = 0.081 and 0.056, respectively; Fig. 4e, h); all other deliverables were not significantly different between MT and non-MT students (Fig. 4c, d, g). Attendance in *Discovery* (*p* = 0.22) was also not significantly different between MT and non-MT students, although MT students did miss significantly less class time (*p* = 0.010) (Fig. 4i, j). Longitudinal assessment of individual deliverables for MT students that participated in three or more *Discovery* terms (Fig. 5) further highlights trend in improvement (Fig. 2f). Greater performance over terms of participation was observed for essay (*p* = 0.0295, Fig. 5a), client meeting (*p* = 0.0003, Fig. 5b), proposal (*p* = 0.0004, Fig. 5c), progress report (*p* = 0.16, Fig. 5d), poster (*p* = 0.0005, Fig. 5e), and presentation (*p* = 0.0295, Fig. 5f) deliverable grades; these trends were all significant with the exception of the progress report (*p* = 0.16, Fig. 5d) owing to strong performance in this deliverable in all terms.

Finally, the expansion of *Discovery* to a second school of lower LOI (i.e., nominally higher aggregate SES) allowed for the assessment of program impact in a new population over 2 terms of programming. A significant (*p* = 0.040) divergence in *Discovery* vs. course grade distribution from the theoretical 1:1 relationship was found in the new cohort (S1 Appendix, Fig. S1), in keeping with the pattern established in this study.

### Teacher perceptions

Qualitative observation in the classroom by high school teachers emphasized the value students independently placed on program participation and deliverables. Throughout the term, students often prioritized *Discovery* group assignments over other tasks for their STEM courses, regardless of academic weight and/or due date. Comparing within this student population, teachers spoke of difficulties with late and incomplete assignments in the regular curriculum but found very few such instances with respect to *Discovery*-associated deliverables. Further, teachers speculated on the good behavior and focus of students in *Discovery* programming in contrast to attentiveness and behavior issues in their school classrooms. Multiple anecdotal examples were shared of renewed perception of student potential; students that exhibited poor academic performance in the classroom often engaged with high performance in this inquiry-focused atmosphere. Students appeared to take a sense of ownership, excitement, and pride in the setting of group projects oriented around scientific inquiry, discovery, and dissemination.

### Student perceptions

**Fig. 6:**
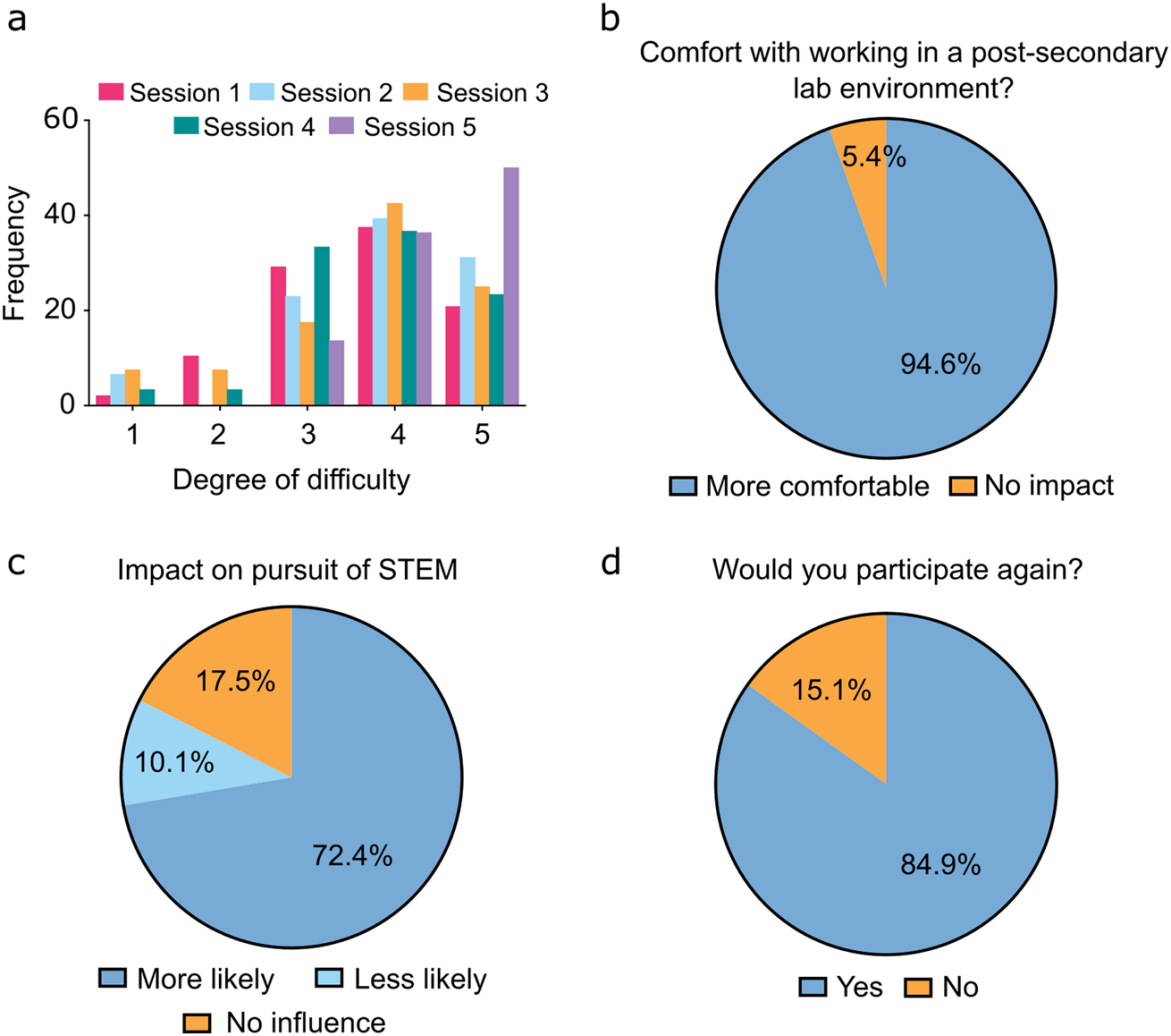
Student survey responses following participation in *Discovery* programming. **a** Histogram of relative frequency of perceived *Discovery* programming academic difficulty ranked from not challenging (1) to highly challenging (5) for each session demonstrated the consistently perceived high degree of difficulty for *Discovery* programming (total responses: 223). **b** Program participation increased student comfort (94.6%) with navigating lab work in a university or college setting (total responses: 220). **c** Considering participation in *Discovery* programming, students indicated their increased (72.4%) or decreased (10.1%) likelihood to pursue future experiences in STEM as a measure of program impact (total responses: 217). **d** Large majority of participating students (84.9%) indicated their interest for future participation in *Discovery* (total responses: 212). Students were given the opportunity to opt out of individual survey questions, partially completed surveys were included in totals.

Students were asked to consider and rank the academic difficulty (scale of 1–5, with 1 = not challenging and 5 = highly challenging) of the work they conducted within the *Discovery* learning model. Considering individual *Discovery* terms, at least 91% of students felt the curriculum to be sufficiently challenging with a 3/5 or higher ranking (Term 1: 87.5%, Term 2: 93.4%, Term 3: 85%, Term 4: 93.3%, Term 5: 100%), and a minimum of 58% of students indicating a 4/5 or higher ranking (Term 1: 58.3%, Term 2: 70.5%, Term 3: 67.5%, Term 4: 69.1%, Term 5: 86.4%) (Fig. 6a).

The majority of students (94.6%) indicated they felt more comfortable with the idea of performing future work in a university STEM laboratory environment given exposure to university teaching facilities throughout the program (Fig. 6b). Students were also queried whether they were (i) more likely, (ii) less likely, or (iii) not impacted by their experience in the pursuit of STEM in the future. The majority of participants (>82%) perceived impact on STEM interests, with 72.4% indicating they were more likely to pursue these interests in the future (Fig. 6c). When surveyed at the end of term, 84.9% of students indicated they would participate in the program again (Fig. 6d).

## Discussion

We have described an inquiry-based framework for implementing experiential STEM education in a BME setting. Using this model, we engaged 268 instances of student participation (170 individual students who participated 1–4 times) over five terms in project-based learning wherein students worked in peer-based teams under the mentorship of U of T trainees to design and execute the scientific method in answering a relevant research question. Collaboration between high school teachers and *Discovery* instructors allowed for high school student exposure to cutting-edge BME research topics, participation in facilitated inquiry, and acquisition of knowledge through scientific discovery. All assessments were conducted by high school teachers and constituted a fraction (10–15%) of the overall course grade, instilling academic value for participating students. As such, students exhibited excitement to learn as well as commitment to their studies in the program.

Through our observations and analysis, we suggest there is value in differential learning environments for students that struggle in a knowledge acquisition-focused classroom setting. In general, we observed a high level of academic performance in *Discovery* programming (Fig. 2a), which was highlighted exceptionally in EE students who exhibited greater academic performance in *Discovery* deliverables compared to normal coursework (>18% grade improvement in relevant deliverables). We initially considered whether this was the result of strong students influencing weaker students; however, group organization within each course suggests this is not the case (Fig. 2d). With the exception of one class in one term (24 participants assigned by their teacher), students were allowed to self-organize into working groups and they chose to work with other students of relatively similar academic performance (as indicated by course grade), a trend observed in other studies^31,32^. Remarkably, EE students not only excelled during *Discovery* when compared to their own performance in class, but this cohort also achieved significantly higher average grades in each of the deliverables throughout the program when compared to the remaining *Discovery* cohort (Fig. 3). This data demonstrates the value of an inquiry-based learning environment compared to knowledge-focused delivery in the classroom in allowing students to excel. We expect that part of this engagement was resultant of student excitement with a novel learning opportunity. It is however a well-supported concept that students who struggle in traditional settings tend to demonstrate improved interest and motivation in STEM when given opportunity to interact in a hands-on fashion, which supports our outcomes^4,33^. Furthermore, these outcomes clearly represent variable student learning styles, where some students benefit from a greater exchange of information, knowledge and skills in a cooperative learning environment^34^. The performance of the EE group may not be by itself surprising, as the identification of the subset by definition required high performers in *Discovery* who did not have exceptionally high course grades; in addition, the final *Discovery* grade is dependent on the component assignment grades. However, the discrepancies between EE and non-EE groups attendance suggests that students were engaged by *Discovery* in a way that they were not by regular classroom curriculum.

In addition to quantified engagement in *Discovery* observed in academic performance, we believe remarkable attendance rates are indicative of the value students place in the differential learning structure. Given the differences in number of *Discovery* days and implications of missing one day of regular class compared to this immersive program, we acknowledge it is challenging to directly compare attendance data and therefore approximate this comparison with consideration of learning time equivalence. When combined with other subjective data including student focus, requests to work on *Discovery* during class time, and lack of discipline/behavior issues, the attendance data importantly suggests that students were especially engaged by the *Discovery* model. Further, we believe the increased commute time to the university campus (students are responsible for independent transit to campus, a much longer endeavour than the normal school commute), early program start time, and students’ lack of familiarity with the location are non-trivial considerations when determining the propensity of students to participate enthusiastically in *Discovery*. We feel this suggests the students place value on this team-focused learning and find it to be more applicable and meaningful to their interests.

Given post-secondary admission requirements for STEM programs, it would be prudent to think that students participating in multiple STEM classes across terms are the ones with the most inherent interest in post-secondary STEM programs. The MT subset, representing students who participated in *Discovery* for more than one term, averaged significantly higher final *Discovery* grades. The increase in the final *Discovery* grade was observed to result from a general confluence of improved performance over multiple deliverables and a continuous effort to improve in a STEM curriculum. This was reflected in longitudinal tracking of *Discovery* performance, where we observed a significant trend of improved performance. Interestingly, the high number of MT students who were included in the EE group suggests that students who had a keen interest in science enrolled in more than one course and in general responded well to the inquiry-based teaching method of *Discovery*, where scientific method was put into action. It stands to reason that students interested in science will continue to take STEM courses and will respond favorably to opportunities to put classroom theory to practical application.

The true value of an inquiry-based program such as *Discovery* may not be based in inspiring students to perform at a higher standard in STEM within the high school setting, as skills in critical thinking do not necessarily translate to knowledge-based assessment. Notably, students found the programming equally challenging throughout each of the sequential sessions, perhaps somewhat surprising considering the increasing number of repeat attendees in successive sessions (Fig. 6a). Regardless of sub-discipline, there was an emphasis of perceived value demonstrated through student surveys where we observed indicated interest in STEM and comfort with laboratory work environments, and desire to engage in future iterations given the opportunity. Although non-quantitative, we perceive this as an indicator of significant student engagement, even though some participants did not yield academic success in the program and found it highly challenging given its ambiguity.

Although we observed that students become more certain of their direction in STEM, further longitudinal study is warranted to make claim of this outcome. Additionally, at this point in our assessment we cannot effectively assess the practical outcomes of participation, understanding that the immediate effects observed are subject to a number of factors associated with performance in the high school learning environment. Future studies that track graduates from this program will be prudent, in conjunction with an ever-growing dataset of assessment as well as surveys designed to better elucidate underlying perceptions and attitudes, to continue to understand the expected benefits of this inquiry-focused and partnered approach. Altogether, a multifaceted assessment of our early outcomes suggests significant value of an immersive and iterative interaction with STEM as part of the high school experience. A well-defined divergence from knowledge-based learning, focused on engagement in critical thinking development framed in the cutting-edge of STEM, may be an important step to broadening student perspectives.

In this study, we describe the short-term effects of an inquiry-based STEM educational experience on a cohort of secondary students attending a non-specialized school, and suggest that the framework can be widely applied across virtually all subjects where inquiry-driven and mentored projects can be undertaken. Although we have demonstrated replication in a second cohort of nominally higher SES (S1 Appendix, Supplementary Fig. 1), a larger collection period with more students will be necessary to conclusively determine impact independent of both SES and specific cohort effects. Teachers may also find this framework difficult to implement depending on resources and/or institutional investment and support, particularly if post-secondary collaboration is inaccessible. Offerings to a specific subject (e.g., physics) where experiments yielding empirical data are logistically or financially simpler to perform may be valid routes of adoption as opposed to the current study where all subject cohorts were included.

As we consider *Discovery* in a bigger picture context, expansion and implementation of this model is translatable. Execution of the scientific method is an important aspect of citizen science, as the concepts of critical thing become ever-more important in a landscape of changing technological landscapes. Giving students critical thinking and problem-solving skills in their primary and secondary education provides value in the context of any career path. Further, we feel that this model is scalable across disciplines, STEM or otherwise, as a means of building the tools of inquiry. We have observed here the value of differential inclusive student engagement and critical thinking through an inquiry-focused model for a subset of students, but further to this an engagement, interest, and excitement across the body of student participants. As we educate the leaders of tomorrow, we suggest that use of an inquiry-focused model such as *Discovery* could facilitate growth of a data-driven critical thinking framework.

In conclusion, we have presented a model of inquiry-based STEM education for secondary students that emphasizes inclusion, quantitative analysis, and critical thinking. Student grades suggest significant performance benefits, and engagement data suggests positive student attitude despite the perceived challenges of the program. We also note a particular performance benefit to students who repeatedly engage in the program. This framework may carry benefits in a wide variety of settings and disciplines for enhancing student engagement and performance, particularly in non-specialized school environments.

## Methods

### Study design and implementation

Participants in *Discovery* include all students enrolled in university-stream Grade 11 or 12 biology, chemistry, or physics at the participating school over five consecutive terms (cohort summary shown in Table 1). Although student participation in educational content was mandatory, student grades and survey responses (administered by high school teachers) were collected from only those students with parent or guardian consent. Teachers replaced each student name with a unique coded identifier to preserve anonymity but enable individual student tracking over multiple terms. All data collected were analyzed without any exclusions save for missing survey responses; no power analysis was performed prior to data collection.

**Table 1:** Summary of cohort participants in each term offering of *Discovery* (*N* = 268 instances of student participation).

Term	Class	Grade	Number of students
1 *N* = 57 students	Biology	11	17
	Chemistry	11	19
		12	4
	Physics	11	17
2 *N* = 63 students	Biology	11	7
		12	18
	Chemistry	11	17
		12	6
	Physics	11	15
3 *N* = 43 students	Biology	11	18
	Chemistry	11	14
	Physics	12	11
4 *N* = 52 students	Biology	11	17
	Chemistry	11	16
	Physics	11	19
5 *N* = 53 students	Biology	12	16
	Chemistry	11	24
		12	6
	Physics	12	7

### Ethics statement

This study was approved by the University of Toronto Health Sciences Research Ethics Board (Protocol # 34825) and the Toronto District School Board External Research Review Committee (Protocol # 2017-2018-20). Written informed consent was collected from parents or guardians of participating students prior to the acquisition of student data (both post-hoc academic data and survey administration). Data were anonymized by high school teachers for maintenance of academic confidentiality of individual students prior to release to U of T researchers.

### Educational program overview

Students enrolled in university-preparatory STEM classes at the participating school completed a term-long project under the guidance of graduate student instructors and undergraduate student mentors as a mandatory component of their respective course. Project curriculum developed collaboratively between graduate students and participating high school teachers was delivered within U of T Faculty of Applied Science & Engineering (FASE) teaching facilities. Participation allows high school students to garner a better understanding as to how undergraduate learning and career workflows in STEM vary from traditional high school classroom learning, meanwhile reinforcing the benefits of problem solving, perseverance, teamwork, and creative thinking competencies. Given that *Discovery* was a mandatory component of course curriculum, students participated as class cohorts and addressed questions specific to their course subject knowledge base but related to the defined global health research topic (Fig. 1). Assessment of program deliverables was collectively assigned to represent 10–15% of the final course grade for each subject at the discretion of the respective STEM teacher.

The *Discovery* program framework was developed, prior to initiation of student assessment, in collaboration with one high school selected from the local public school board over a 1.5 year period of time. This partner school consistently scores highly (top decile) in the school board’s Learning Opportunities Index (LOI). The LOI ranks each school based on measures of external challenges affecting its student population therefore schools with the greatest level of external challenge receive a higher ranking^35^. A high LOI ranking is inversely correlated with socioeconomic status (SES); therefore, participating students are identified as having a significant number of external challenges that may affect their academic success. The mandatory nature of program participation was established to reach highly capable students who may be reluctant to engage on their own initiative, as a means of enhancing the inclusivity and impact of the program. The selected school partner is located within a reasonable geographical radius of our campus (i.e., ~40 min transit time from school to campus). This is relevant as participating students are required to independently commute to campus for *Discovery* hands-on experiences.

Each program term of *Discovery* corresponds with a five-month high school term. Lead university trainee instructors (3–6 each term) engaged with high school teachers 1–2 months in advance of high school student engagement to discern a relevant overarching global healthcare theme. Each theme was selected with consideration of (a) topics that university faculty identify as cutting-edge biomedical research, (b) expertise that *Discovery* instructors provide, and (c) capacity to showcase the diversity of BME. Each theme was sub-divided into STEM subject-specific research questions aligning with provincial Ministry of Education curriculum concepts for university-preparatory Biology, Chemistry, and Physics^9^ that students worked to address, both on-campus and in-class, during a term-long project. The *Discovery* framework therefore provides students a problem-based learning experience reflective of an engineering capstone design project, including a motivating scientific problem (i.e., global topic), subject-specific research question, and systematic determination of a professional recommendation addressing the needs of the presented problem.

*Discovery* instructors were volunteers recruited primarily from graduate and undergraduate BME programs in the FASE. Instructors were organized into subject-specific instructional teams based on laboratory skills, teaching experience, and research expertise. The lead instructors of each subject (the identified 1–2 trainees that built curriculum with high school teachers) were responsible to organize the remaining team members as mentors for specific student groups over the course of the program term (~1:8 mentor to student ratio).

All *Discovery* instructors were familiarized with program expectations and trained in relevant workspace safety, in addition to engagement at a teaching workshop delivered by the Faculty Advisor (a Teaching Stream faculty member) at the onset of term. This workshop was designed to provide practical information on teaching and was co-developed with high school teachers based on their extensive training and experience in fundamental teaching methods. In addition, group mentors received hands-on training and guidance from lead instructors regarding the specific activities outlined for their respective subject programming (an exemplary term of student programming is available in S2 Appendix).

*Discovery* instructors were responsible for introducing relevant STEM skills and mentoring high school students for the duration of their projects, with support and mentorship from the Faculty Mentor. Each instructor worked exclusively throughout the term with the student groups to which they had been assigned, ensuring consistent mentorship across all disciplinary components of the project. In addition to further supporting university trainees in on-campus mentorship, high school teachers were responsible for academic assessment of all student program deliverables (Fig. 1; the standardized grade distribution available in S3 Appendix). Importantly, trainees never engaged in deliverable assessment; for continuity of overall course assessment, this remained the responsibility of the relevant teacher for each student cohort.

Throughout each term, students engaged within the university facilities four times. The first three sessions included hands-on lab sessions while the fourth visit included a culminating symposium for students to present their scientific findings (Fig. 1). On average, there were 4–5 groups of students per subject (3–4 students per group; ~20 students/class). *Discovery* instructors worked exclusively with 1–2 groups each term in the capacity of mentor to monitor and guide student progress in all project deliverables.

After introducing the selected global research topic in class, teachers led students in completion of background research essays. Students subsequently engaged in a subject-relevant skill-building protocol during their first visit to university teaching laboratory facilities, allowing opportunity to understand analysis techniques and equipment relevant for their assessment projects. At completion of this session, student groups were presented with a subject-specific research question as well as the relevant laboratory inventory available for use during their projects. Armed with this information, student groups continued to work in their classroom setting to develop group-specific experimental plans. Teachers and *Discovery* instructors provided written and oral feedback, *respectively*, allowing students an opportunity to revise their plans in class prior to on-campus experimental execution.

Once at the relevant laboratory environment, student groups executed their protocols in an effort to collect experimental data. Data analysis was performed in the classroom and students learned by trial & error to optimize their protocols before returning to the university lab for a second opportunity of data collection. All methods and data were re-analyzed in class in order for students to create a scientific poster for the purpose of study/experience dissemination. During a final visit to campus, all groups presented their findings at a research symposium, allowing students to verbally defend their process, analyses, interpretations, and design recommendations to a diverse audience including peers, STEM teachers, undergraduate and graduate university students, postdoctoral fellows and U of T faculty.

### Data collection

Teachers evaluated their students on the following associated deliverables: (i) global theme background research essay; (ii) experimental plan; (iii) progress report; (iv) final poster content and presentation; and (v) attendance. For research purposes, these grades were examined individually and also as a collective *Discovery* program grade for each student. For students consenting to participation in the research study, all *Discovery* grades were anonymized by the classroom teacher before being shared with study authors. Each student was assigned a code by the teacher for direct comparison of deliverable outcomes and survey responses. All instances of “Final course grade” represent the prorated course grade without the *Discovery* component, to prevent confounding of quantitative analyses.

Survey instruments were used to gain insight into student attitudes and perceptions of STEM and post-secondary study, as well as *Discovery* program experience and impact (S4 Appendix). High school teachers administered surveys in the classroom only to students supported by parental permission. Pre-program surveys were completed at minimum 1 week prior to program initiation each term and exit surveys were completed at maximum 2 weeks post-*Discovery* term completion. Surveys results were validated using a principal component analysis (S1 Appendix, Supplementary Fig. 2).

### Identification and comparison of population subsets

From initial analysis, we identified two student subpopulations of particular interest: students who performed ≥1 SD [18.0%] or greater in the collective *Discovery* components of the course compared to their final course grade (“EE”), and students who participated in *Discovery* more than once (“MT”). These groups were compared individually against the rest of the respective *Discovery* population (“non-EE” and “non-MT”, *respectively*). Additionally, MT students who participated in three or four (the maximum observed) terms of *Discovery* were assessed for longitudinal changes to performance in their course and *Discovery* grades. Comparisons were made for all *Discovery* deliverables (introductory essay, client meeting, proposal, progress report, poster, and presentation), final *Discovery* grade, final course grade, *Discovery* attendance, and overall attendance.

### Statistical analysis

Student course grades were analyzed in all instances without the *Discovery* contribution (calculated from all deliverable component grades and ranging from 10 to 15% of final course grade depending on class and year) to prevent correlation. Aggregate course grades and *Discovery* grades were first compared by paired t-test, matching each student’s course grade to their *Discovery* grade for the term. Student performance in *Discovery* (*N* = 268 instances of student participation, comprising 170 individual students that participated 1–4 times) was initially assessed in a linear regression of *Discovery* grade vs. final course grade. Trends in course and *Discovery* performance over time for students participating 3 or 4 terms (*N* = 16 and 3 individuals, *respectively*) were also assessed by linear regression. For subpopulation analysis (EE and MT, *N* = 99 instances from 81 individuals and 174 instances from 76 individuals, *respectively*), each dataset was tested for normality using the D’Agostino and Pearson omnibus normality test. All subgroup comparisons vs. the remaining population were performed by Mann–Whitney *U*-test. Data are plotted as individual points with mean ± SEM overlaid (grades), or in histogram bins of 1 and 4 days, *respectively*, for *Discovery* and class attendance. Significance was set at α ≤ 0.05.

### Reporting summary

Further information on research design is available in the Nature Research Reporting Summary linked to this article.

## Supplementary information

Supplemental Material

Reporting Summary

## Data Availability

The data that support the findings of this study are available upon reasonable request from the corresponding author DMK. These data are not publicly available due to privacy concerns of personal data according to the ethical research agreements supporting this study.
